# Disparities in the medical expenditures of patients with cancer and concomitant mental disorder: analyzing the effects of diagnosis sequence order

**DOI:** 10.1186/s12913-023-09056-9

**Published:** 2023-01-27

**Authors:** Kai-Jie Ma, Shu-Yuan Su, Daniel Nie, Wei-Sheng Chung, Chi-Yu Yao, Jong-Yi Wang

**Affiliations:** 1 Department of Public Health, China Medical University, Taichung, Taiwan; 2 Department of Health Services Administration, China Medical University, Taichung, Taiwan; 3 Department of Internal Medicine, Taichung Hospital, Taichung, Taiwan; 4 Department of Healthcare Administration, Central Taiwan University of Science and Technology, Taichung, Taiwan; 5 Attending physician Department of psychiatry, An-nan hospital, Tainan, Taiwan

**Keywords:** Cancer, Mental disorder, Diagnosis sequence, Medical expenditures, High-utilization

## Abstract

**Background:**

Cancer is the leading cause of death in Taiwan. Medical expenditures related to cancer accounted for 44.8% of all major illness insurance claims in Taiwan. Prior research has indicated that the dual presence of cancer and mental disorder in patients led to increased medical burden. Furthermore, patients with cancer and concomitant mental disorder could incur as much as 50% more annual costs than those without. Although previous studies have investigated the utilization of patients with both diseases, the effects of morbidity sequence order on patient costs are, however, uncertain. This study explored medical expenditures linked with the comorbidity of cancer and mental disorder, with a focus on the impact of diagnosis sequence order.

**Methods:**

This population-based retrospective matched cohort study retrieved patients with cancer and mental disorder (aged ≥ 20 years) from the Ministry of Health and Welfare Data Science Center 2005–2015 database. 321,045 patients were divided based on having one or both diseases, as well as on the sequence of mental disorder and cancer diagnosis. Study subjects were paired with comparison counterparts free of both diseases using Propensity Score Matching at a 1:1 ratio. Annual Cost per Patient Linear Model (with a log-link function and gamma distribution) was used to assess the average annual cost, covarying for socio-demographic and clinical factors. Binomial Logistic Regression was used to evaluate factors associated with the risk of high-utilization.

**Results:**

The “Cancer only” group had higher adjusted mean annual costs (NT$126,198), more than 5-times that of the reference group (e^β: 5.45, *p* < 0.001). However, after exclusion of patients with non-cancer and inclusion of diagnosis sequence order for patients with cancer and concomitant mental disorder, the post-cancer mental disorder group had the highest expenditures at over 13% higher than those diagnosed with only cancer on per capita basis (e^β: 1.13, *p* < 0.001), whereas patients with cancer and any pre-existing mental disorder incurred lower expenditures than those with only cancer. The diagnosis of post-cancer mental disorder was significantly associated with high-utilization (OR = 1.24; 95% CI: 1.047–1.469). Other covariates associated with high-utilizer status included female sex, middle to old age, and late stage cancer.

**Conclusion:**

Presence of mental disorder prior to cancer had a diminishing effect on medical utilization in patients, possibly indicating low medical compliance or adherence in patients with mental disorder on initial treatments after cancer diagnosis. Patients with post-cancer mental disorder had the highest average annual cost. Similar results were found in the odds of reaching high-utilizer status. The follow-up of cancer treatment for patients with pre-existing mental disorders warrants more emphasis in an attempt to effectively allocate medical resources.

**Supplementary Information:**

The online version contains supplementary material available at 10.1186/s12913-023-09056-9.

## Introduction

Healthcare expenditures continue to grow at a fast pace around the world. U.S. National Health Expenditures grew5% annually from 2005 to 2017, and stands at 3.3 trillion dollars or 17.9% of GDP [1]. US health care spending increased 4.6 percent to reach $3.8 trillion in 2019, similar to the rate of growth of 4.7 percent in 2018. The share of the economy devoted to health care spending was 17.7 percent in 2019 compared with 17.6 percent in 2018 [2, 3]. Conditions in Taiwan are similar: national healthcare expenditures have increased at an average annual rate of 3.6% [4]. Meanwhile, NHI revenue has not kept pace with the growth in expenditures. In 2017, NHI expenses grew 5.5% while revenue only increased 0.5%, and in 2016 revenue actually fell 8.4% while expenses grew 5.6% [4]. Furthermore, over half of all major illness inpatient expenditures in Taiwan is spent for the treatment of just two diseases: cancer(46%) and mental disorder (MD) (11%) [4].

Cancer is the second leading cause of death, and is estimated to be responsible for 9.8 million deaths in 2018or 16.7% of all deaths worldwide [5]. In Taiwan, it ranks as the number 1 cause of mortality, where annual cancer deaths currently account for 28.2% of all deaths [6].The World Health Organization(WHO) also states “the number of new cases is expected to rise by about 70% over the next 2 decades”. Description of past research that individuals are likely to simultaneously suffer from both malignant neoplasms and mental illness. Psychiatric issues were found present in 20%, up to as much as 50%, of cancer patients [7–10]. These study findings suggest that the prevalence of mental disorder among cancer cohorts is consistently much higher than in the general population. Mental disorders encompass different illnesses that are defined by the WHO as being characterized by “abnormal thoughts, perceptions, emotions, behavior and relationships with others.” The WHO also states that individuals with either depression or schizophrenia have a 40 to 60% higher chance of premature death as compared to the general population [5] caused by somatic illnesses such as cancer. In Taiwan, the prevalence of mental illness has risen from 8.8% just ten years ago to 11% in 2016 and is expected to continue to rise [6–11].

The Organisation for Economic Co-operation and Development estimates that cancer, mental health, along with circulatory disease, account for over 40% of all hospital spending, and “cancer is the second most important disease group accounting for around 13% of hospital spending [12].The total annual economic cost of cancer was estimated to be US$ 1.16 trillion in the WHO’s Annual Cancer Report 2018 [5]. In Taiwan, the medical utilization of cancer occupies nearly 40% of all major illness insurance claims (Outpatient: 33.6%, Inpatient: 45.7%) [6]. The dual presence of both cancer and mental disorder in patients leads to increased more expenditures. Past research has shown a disparity in the monetary burden between cancer patients with poor mental health and those without. The increase can be as large as24% to over 50% [13–15]. These large and adverse effects of comorbidity with mental disease merits further research involving cost analysis.

Cancer patients have been shown to be more likely to develop psychiatric disorders [16–18], and the combined cost of cancer with concomitant mental disorder is not an additive equation, but an exponential one [19]. Due to the heavy weighting credited to cancer and mental illness in the profile of healthcare expenditures [4], it would be worthwhile to investigate the costs associated with these disorders [20–23]. Despite the growing importance, there remains a lack of research looking at the intersection of cancer and psychiatric disorder. There has especially been scarce research dividing the comorbid cohort by sequence of diagnosis and conducting a directional analysis. This study attempts to close the knowledge gap in researching the effects of the temporal order of the onset of these two diseases on patient utilization. It’s also one of the first cost analysis studies involving these two important diseases with propensity score matching to pair the study cohort with a comparison cohort. With the depth of information available in the databases of the Taiwan Ministry of Health and Welfare, insights extracted could prove beneficial in enacting policies for resource reallocation, and potentially work toward reducing the economic burden on already strained national funds. Especially as healthcare delivery administrators try to strike a balance between patients’ prolonged survival, quality of life, and medical expenditures [24].

## Materials and methods

Our research is using National Health Insurance Research Database (NHIRD) from the Ministry of Health and Welfare (MOHW) Data Science Center 2005–2015, this retrospective matched-cohort study retrieves patients with cancer and/or mental disorder aged over 20 years. Target patients are divided into 3 groups based on diagnosis of either cancer or MD or both. All patients are then matched with a counterpart free of both cancer and MD, forming the comparison counterpart group. A second framework involving cancer patients with concomitant MD compares different diagnosis sequence order groups with cancer patients without MD.

### Data sources

Established in 1995, NHIRD is the most complete electronic health record in Taiwan. The NHIRD covers more than 99.6% of the Taiwanese population, and contains demographic variables, outpatient and inpatient information, prescriptions, diagnosis information, medical personnel information and other detailed clinical information [25]. Our data sources were six types of registration files, Registry for Beneficiaries, Ambulatory Care Expenditures, Inpatient Expenditures by Admissions, Personal Attribute file, Hospital Assessment file and Cancer Registry file. Our study was approved by the Research Ethics Committee of China Medical University Hospital, Taiwan, for protect the patients’ privacy, all personal identification numbers were encrypted by the National Health Research Institutes before the data were released. The Taiwan National Health Research Institutes encrypts patient personal information to protect privacy and provides researchers with anonymous scrambled identification numbers associated with relevant diseases information. Therefore, patient informed consent is not required for authorized researchers to access this research database.

### Inclusion and exclusion criteria

Our study retrieves subjects and inclusion with cancer from the “Cancer Registry File” (diagnostic code ICD-9-CM: 140.xx to 208.xx excluding Kaposi sarcoma, and gender specific genital cancers) and with mental disorder using diagnostic code 290.xx to 319.xx. Main exclusion criteria include the following: Events occurring in the first 2 years of the data set (2005–2006), due to the inability to determine first instance of diagnosis; Patients with unconfirmed diagnosis, i.e. only1diagnosis; Minors below the age of 20; Patients with missing data or examining the average annual cost per patient (ACPP), and high-utilizer analysis, patients without a full year of cost data was excluded in order to ensure a uniform observation period. Therefore, an additional exclusion criterion was included: Disease duration less than 1 year (from diagnosis to present date or death). Lastly, propensity score matching (PSM) is used to match subjects from each case cohort with control counterparts (free of both cancer and mental disorder) on a 1:1 ratio. Matching was done based on the following 5 variables: sex, age, comorbidity index, salary-based premium, and level of urbanization. The detailed study population flow chart can be found in Figure S1.

### High-utilizers

The dual presence of both diseases often leads to patients being in the upper 10% of medical cost spenders, labeled as “high utilizers” (HU). Robinson et al. (2016) found that this group in the top tenth percentile utilize 40% of total annual costs, and that HUs on average incur 4.4 to 9.7 times the cost of non-HUs. In a Medicare-based study, it was found that high-cost patients were much more likely to have a mental health diagnosis (16.0% vs. 6.4%), and among patients segmented as “chronic”, the top 10% of spenders were likely cancer patients [26, 27].

### Charlson comorbidities index

Our research used the Charlson Comorbidity Index (CCI) developed by Charlson in 1984 to evaluate the mortality risk and burden of disease, address the confounding influence of comorbidities, and predict outcomes. We followed the method proposed by Charlson; the CCI consists of 17 comorbidities, weighted from 1 to 6 according to mortality risk and disease severity, and then summed scores to form the total CCI score [28]. However, subjects rarely displayed high CCI scores in our research, so we divided the CCI categories into three groups as follows: 0-1point, 2 point, 3 point and 4 or above points.

### Statistical analysis

Our research used descriptive statistics, namely frequencies and percentages, to understand the sample distribution with respect to each variable. Means and standard deviations were used to represent continuous data which are average annual costs per capita and end-of-life costs. All cost data is discounted forward to 2018 dollars at a rate of 2% per annum. 2% was chosen as it most closely resembles Taiwan inflation rates averaged over the period from year 2005 to 2018 [29]. Because cost data is generally non-normal and right-skewed, Generalized Linear Model (GLM) with a log-link function (providing multiplicative instead of additive covariate effects) and gamma distribution is used to conduct multivariate analysis. The model outputs parameter estimates for categorical independent variables, which can be exponentiated to compare and show the ratio of the costs of one variable group to that of another group. i.e.:

$${e}^{\beta }= \frac{{\widehat{y}}_{1}}{{\widehat{y}}_{c}}$$

where β is the variable coefficient, ${\widehat{y}}_{1}$ is the estimated sample average of group 1 and ${\widehat{y}}_{c}$ is the estimated average of the reference group. In other words, if the exponent of β1 = 1.5, then the cost of those in the target group is 1.5 times, or 50% higher than, that of the reference group. In addition to cost ratios, the model also provides estimated mean costs for each target cohort. The intercept and the variable coefficient summed and exponentiated to directly arrive at estimated mean expenditure for that group. Statistical significance is set at α = 0.05.

Lastly, binomial logistic regression was used to interpret data between High-Utilizers and non-HUs. Patient factors, such as belonging to which sequence order subgroup and other characteristics, was tested for likelihood of HU status. To detect collinearity, the variance inflation factor (VIF) and tolerance of all predictor variables was calculated. All these analyses were conducted using SPSS 22 software.

## Results

The extracted case cohort included 321,045 patients that were diagnosed with either cancer, mental disorder, or both. The case cohort was matched with comparison counterparts for a total of 642,178 patients (Figure S1). Within the case cohort, 301,555 had mental disorder, 11,963 had cancer, and 7,527 had both. Further dividing by sequence order,2,498 patients were diagnosed with pre and post-cancer MD, 2,847 with pre-cancer MD, and 2,182post-cancer MD. Among cancer patients the prevalence of mental disorder was 39% (Table S1). In addition, the majority were female (55.3%) in the study samples. Approximately 24% was aged above 60 years old, and the largest age group was in the 40 to 49 years bracket (23.0%). Most were of married status (79%) and more than half did not attain an education of above high-school level (59%). Majority percentage of patients had premium-based salaries below NT$20,008 per month (63%), and fell within occupation category 1 (44%) living in either the Taipei or Central region (56% combined). Patients within urbanization levels 1 and 2 also combined for over 57% of all patients (Table 1).

**Table 1:** Patient sociodemographic characteristics

Variable	Case Cohort			Comparison Cohort		*P*
	**no**	**%**		**no**	**%**	
**Sex**						1
Male	143,457	44.70%		143,485	44.70%	
Female	177,588	55.30%		177,648	55.30%	
**Age**						1
20–29 years	43,811	13.60%		43,824	13.60%	
30–39 years	60,678	18.90%		60,683	18.90%	
40–49 years	73,751	23.00%		73,779	23.00%	
50–59 years	66,025	20.60%		66,048	20.60%	
60–69 years	38,831	12.10%		38,844	12.10%	
over 70 years	37,949	11.80%		37,955	11.80%	
**Marital Status**						< 0.01
Single	64,655	20.10%		66,620	20.80%	
Married	256,390	79.90%		254,425	79.20%	
**Education Level**						< 0.01
Middle school or Below	133,812	41.70%		130,236	40.60%	
Highschool	102,309	31.90%		99,240	30.90%	
College or Above	84,924	26.50%		91,569	28.50%	
**Premium-based Salary**						1
$20,008 or below	204,754	63.80%		204,805	63.80%	
$20,009–28,800	28,934	9.00%		28,940	9.00%	
$28,801–45,800	54,994	17.10%		55,016	17.10%	
$45,801 or above	32,363	10.10%		32,372	10.10%	
**Occupation**						< 0.01
Category 1	139,703	43.50%		143,596	44.70%	
Category 2	57,393	17.90%		57,298	17.80%	
Category 3	53,612	16.70%		55,668	17.30%	
Category 4/6	67,315	21.00%		62,548	19.50%	
Category 5	3,022	0.90%		1,935	0.60%	
**Geographic Region**						< 0.01
Taipei	114,379	35.60%		116,335	36.20%	
Northern	36,674	11.40%		39,157	12.20%	
Central	64,430	20.10%		59,816	18.60%	
Southern	44,567	13.90%		44,368	13.80%	
Kaohsiung/Ping Tung	52,951	16.50%		52,912	16.50%	
Eastern	8,044	2.50%		8,457	2.60%	
**Level of Urbanization**						1
1 (highest)	89,445	27.90%		89,476	27.90%	
2	93,825	29.20%		93,857	29.20%	
3	54,511	17.00%		54,520	17.00%	
4	47,272	14.70%		47,278	14.70%	
5	6,661	2.10%		6,662	2.10%	
6	14,134	4.40%		14,138	4.40%	
7 (lowest)	15,197	4.70%		15,202	4.70%	
**CCI**						1
0–1	227,629	70.90%		227,718	70.90%	
2	47,096	14.70%		47,102	14.70%	
3	23,679	7.40%		23,677	7.40%	
4 or above	22,641	7.10%		22,636	7.10%	
**Hospital Level**						< 0.01
Medical Center	59,128	18.40%	23,267		7.20%	
Regional	75,257	23.40%	23,541		7.30%	
District	35,456	11.00%	18,547		5.80%	
Clinic	151,204	47.10%	255,690		79.60%	
**Hospital Ownership**						< 0.01
Public	60,078	18.70%	15,733		4.90%	
Private	181,144	56.40%	262,141		81.70%	
Consortium	73,508	22.90%	41,787		13.00%	
Association	6,315	2.00%	1,384		0.40%	

### Average annual cost per patient

We used GLM to analyze cancer, MD patients’ costs (Table 2), found that without incorporating diagnose sequence considerations between cancer and MD, the average annual expenditure of cancer patients without any mental disorder diagnosis is more than five-fold that of the reference group (e^β = 5.45, *p*-value: < 0.001). Patients with both cancer and MD incur annual expenditures that are 3.5-times higher than reference group patients (e^β = 3.55, *p*-value: < 0.001), The mental disorder only cohort had slightly higher average annual costs, at 1.59 times the cost of the reference cohort. In addition, females incur annual costs 1.04 times higher than males. Using the lowest age group as reference, those aged 60–69 years and over 70 years incur more than double the cost, while those in the 50–59 age group incur 77% more costs. Married patients have slightly reduced average annual expenditures than single patients (e^β = 0.92, *p*-value: < 0.001). As for education level, with the lowest level (middle school or below) as reference, the high-school group had a very small significant difference (e^β = 0.99, *p*-value: 0.049). The average annual expenditure of college or above group is less than five-fold that of the reference group (e^β = 0.92, *p*-value: < 0.001). The group of patients in the lowest premium-based salary bracket had the lowest costs but differences were very small between brackets (e^β = 1.02 to 1.03). Cohort geographic region, residence level of urbanization and occupation also resulted in very small significant differences (e^β = 0.91 to 1.31). The CCI groups of patients with levels of 2, 3 and 4 or above incurred annual costs at over 1.75, 2.39 and 4.44 times the costs of the reference group (CCI level 0–1). Finally, Medical Center hospitals and private hospitals received patients incurring the highest average cost per year.

**Table 2:** Effect of diagnosis and other covariates on expenditures

Variable	e^β	*p*-value	est. Mean
**Cohort**			
Cancer & MD	3.55	< .001	112,757
Cancer only	5.45	< .001	173,349
MD only	1.59	< .001	50,435
Free of both	1	––	31,802
**Sex**			
Female	1.04	< .001	76,193
Male	1	––	73,486
**Age**			
20–29 years	1	––	46,736
30–39 years	1.15	< .001	53,831
40–49 years	1.4	< .001	65,332
50–59 years	1.77	< .001	82,701
60–69 years	2.36	< .001	110,107
over 70 years	2.51	< .001	117,281
**Marital Status**			
Single	1	––	78,108
Married	0.92	< .001	71,685
**Education Level**			
Middle school or below	1		75,669
High school	0.99	0.049	75,173
College or above	0.97	< .001	73,655
**Premium-based Salary**			
$20,008 or below	1	––	73,505
$20,009–28,800	1.03	< .001	75,953
$28,801–45,800	1.02	< .001	74,722
$45,801 or above	1.02	< .001	75,150
**Occupation**			
Category 1	1	––	70,592
Category 2	1.06	< .001	74,978
Category 3	0.96	< .001	67,932
Category 4/6	1	0.94	70,611
Category 5	1.31	< .001	92,399
**Geographic Region**			
Taipei	1	––	72,291
Northern	0.99	0.004	71,381
Central	1.08	< .001	77,745
Southern	1.05	< .001	76,228
Kaohsiung	1.05	< .001	75,637
Eastern	1.05	< .001	75,889
**Level of Urbanization**			
1 (highest)	1	––	78,591
2	0.97	< .001	75,950
3	0.98	< .001	77,320
4	0.95	< .001	74,712
5	0.91	< .001	71,862
6	0.91	< .001	71,503
7 (lowest)	0.94	< .001	74,133
**Charlson Comorbidity Index**			
0–1	1	––	36,030
2	1.75	< .001	63,129
3	2.39	< .001	86,141
4 or above	4.44	< .001	160,007
**Hospital Level**			
Medical Center	1	––	87,755
Regional	0.87	< .001	76,309
District	0.82	< .001	71,806
Clinic	0.74	< .001	65,197
**Hospital Ownership**			
Public	1	––	71,612
Private	1.05	< .001	75,547
Consortium	1.01	0.234	72,044
Association	1.12	< .001	80,434

Results of the effects of cancer & MD diagnosis sequence, after adjusting for cancer type, cancer stage, and other covariates on annual expenditures using GLM (Table 3). The cancer group without MD was the reference group and had the second highest average ACPP. Those with a post-cancer MD diagnosis incurred the highest average annual costs spending over 13% more than the cancer only group (e^β = 1.13, *p*-value: < 0.001). The two other groups, with pre- & post-cancer MD diagnosis, and pre-cancer MD diagnosis, both incurred lower average annual costs as compared to the reference group (e^β = 0.60, *p*-value: < 0.001; e^β = 0.46, *p*-value: < 0.001). The pre-cancer MD group had the lowest costs, at less than half that of the reference group.

**Table 3:** Effect of diagnosis and cancer covariates on expenditures

Variable	e^β	*p*-value	est. Mean
**Cohort**			
Pre & Post-Cancer MD	0.60	< .001	65,752
Pre-Cancer MD	0.46	< .001	51,163
Post-Cancer MD	1.13	< .001	124,591
Cancer Only**(ref)**	1	––	110,160

### Cancer cohort high-utilizer

Out of the total 19,490 study subjects with a cancer diagnosis, the upper 10% of cost utilizers accounted for 41% of all expenditures. The group with post-cancer MD had the highest percentage of HU: 13%. The cancer only group had the second highest at 9% with HU status. The other 2 groups, pre & post-cancer MD and pre-cancer MD, had the least patients classified as HU (S3). To detect collinearity, the VIF and tolerance of predictor variables was calculated. Variance inflation factors ranged from 1.01958 to 1.58202, well below the accepted level of 10, while the minimum tolerance was 0.6321. Cancer patients with a concomitant MD diagnosis prior to cancer was significantly associated with decreased odds of HU status under adjusted binomial logistic regression (Table 4). MD diagnosis post-cancer was significantly associated with increased odds of HU status. Compared to the reference group (Cancer Only) those with both pre & post-cancer MD diagnosis and those with pre-cancer MD diagnosis had the lowest association with high-utilization (aOR: 0.191 [95% CI: 0.141—0.258], aOR: 0.082 [95% CI: 0.056—0.120], respectively). The post-cancer MD group had an adjusted odds ratio of 1.240 (95% CI: 1.047—1.469). In addtiion, liver and bile duct cancers, colorectal cancer, and oral cancer were all significantly associated with lowered risk of HU status as compared to other cancers. Lung cancer and breast cancer patients had significantly higher risk of being a HU (aOR: 2.139 [95% CI: 1.808- 2.532]; AOR: 1.431 [95% CI: 1.173–1.746]). Later cancer stage was significantly associated with higher HR risk. Other covariates significantly associated with high-utilizer status include the female sex (aOR: 0.819, 95% CI: 0.712- 0.942), and middle to old age from 30 to 69 years old (aOR: 2.330 to 2.704). CCI level3 was significantly associated with lowered risk of HU status as compared to CCI of 2, although the difference was small (aOR: 0.803, 95% CI: 0.669- 0.965), while CCI of 4 or above did not have significantly different risks of HU.

**Table 4:** Predictors of cancer cohort HU status

Variable	cOR	95% CI	aOR	95% CI
**Cohort**				
Pre & Post-Cancer MD	0.171**	0.129—0.227	0.191**	0.141—0.258
Pre-Cancer MD	0.089**	0.062—0.127	0.082**	0.056—0.120
Post-Cancer MD	0.945	0.814—1.096	1.240*	1.047—1.469
Cancer Only**(ref)**	1	––	1	––
**Sex**				
Female **(ref)**	1	––	1	––
Male	0.783**	0.707- 0.867	0.819**	0.712—0.942
**Age**				
20–29 years **(ref)**	1	––	1	––
30–39 years	2.480**	1.241—4.950	2.704**	1.798—5.549
40–49 years	2.398**	1.221—4.712	2.563**	1.872—5.202
50–59 years	2.696**	1.377—5.278	2.696**	1.932—5.481
60–69 years	2.416**	1.231—4.744	2.330**	1.969—4.766
over 70 years	1.906*	1.014—3.746	1.487	0.723—3.059
**Marital Status**				
Single **(ref)**	1	––	1	––
Married	0.772*	0.656—0.908	0.86	0.712—1.038
**Education Level**				
Middle school or Below	0.921	0.806—1.051	1.176	0.991—1.395
Highschool	1.003	0.864—1.166	1.074	0.909—1.270
College or Above	1	–-	1	––
**Premium-based Salary**				
$20,008 or below	0.914	0.934—1.514	0.978	0.778—1.230
$20,009–28,800	1.340*	0.798—1.425	1.266	0.977—1.639
$28,801–45,800	1.164	0.631—1.146	1.164	0.945—1.435
$45,801 or above **(ref)**	1	––	1	––
**Occupation**				
Category 1 **(ref)**	1	––	1	––
Category 2	1.21	1.021—1.433	1.073	0.879—1.309
Category 3	0.962*	0.811—1.141	1.118	0.914—1.369
Category 4/6	1.376**	1.192—1.589	1.222*	1.005—1.485
Category 5	1.502	0.772—2.924	1.561	0.753—3.237
**Geographic Region**				
Taipei **(ref)**	1	––	1	––
Northern	0.929	0.777—1.110	1.069	0.871—1.313
Central	1.195*	1.042—1.371	1.117	0.867—1.682
Southern	0.847*	0.724—0.995	1.173	0.967—1.423
Kaohsiung Pingtung	0.792*	0.676—0.926	0.864	0.723—1.033
Eastern	0.968	0.703—1.333	1.271	0.888—1.818
**Level of Urbanization**				
1 (highest) **(ref)**	1	––	1	––
2	0.837*	0.732—0.958	0.800*	0.685—0.935
3	0.953	0.769—1.109	0.879	0.733—1.054
4	0.85	0.711—1.001	0.822	0.669—1.010
5	0.571*	0.870—0.829	0.640*	0.417—0.984
6	0.582*	0.822—0.785	0.584*	0.584—0.833
7 (lowest)	0.99	1.093—1.244	1.093	0.837—1.426
**CCI**				
2 **(ref)**	1	––	1	––
3	0.768*	0.644—0.916	0.803*	0.669—0.965
4 or above	0.790*	0.686—0.911	0.902	0.769—1.058
**Cancer Type**				
Lung and Bronchus	2.018**	1.219—1.821	2.139**	1.808—2.532
Liver and Bile Duct	0.690**	0.584—0.887	0.812*	0.665—0.992
Colorectal	0.531**	0.695—1.005	0.561**	0.459—0.685
Breast	1.212*	0.266—0.455	1.431**	1.173—1.746
Oral Cavity	0.685**	1.157—1.717	0.669**	0.548—0.818
Others **(ref)**	1	––	1	––
**Cancer Stage**				
Stage 0-I**(ref)**	1	––	1	
Stage II	1.994**	1.685—2.361	2.018**	1.690—2.408
Stage III	2.608**	2.212—3.074	3.236**	2.713—3.859
Stage IV	3.445**	2.954—4.017	3.529**	2.979—4.181
**Hospital Level**				
Medical Center	3.020**	2.513—3.628	1.228	0.956—1.577
Regional	2.215**	1.827—2.686	0.906	0.702—1.171
District	1.638**	1.229—2.185	1.067	0.776—1.467
Clinic **(ref)**	1	––	1	––
**Hospital Ownership**				
Public **(ref)**	1	––	1	––
Private	0.697**	0.605—0.805	1.103	0.925—1.315
Consortium	1.092	0.965—1.235	1.14	0.998—1.302
Association	1.074	0.809—1.426	1.156	0.838—1.594

^*^p-value < 0.05

^**^p-value < 0.001

### End of life costs

**Fig. 1:**
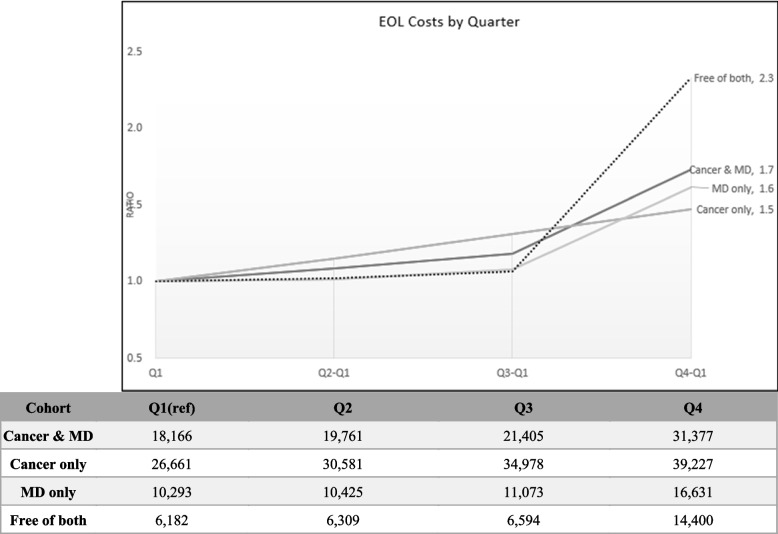
Median EOL Costs by Quarter

**Fig. 2:**
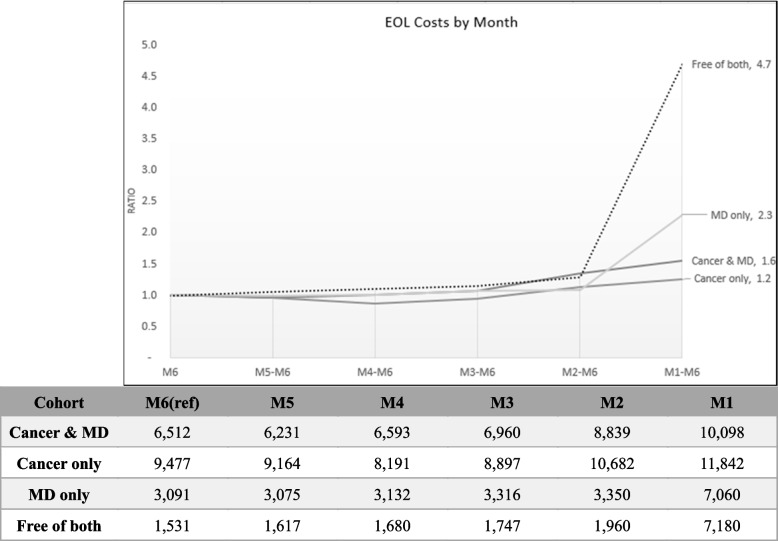
Median EOL Costs by Month

At the end-of-life (EOL), the median quarterly cost of patients free of both disorders increased at the fasted pace among cohorts, where final quarter costs were 2.3 times higher than the first quarter (Fig. 1). Followed by the Cancer & MD groups, and the MD only group, while the cancer only group had the slowest increases in quarterly EOL costs. In addition, the same results were seen for EOL monthly costs during the final half year of life of patients, as shown in Fig. 2. Patient’s free of both disorders increased at the fasted pace among cohorts, where final quarter costs were 4.7 times higher than the first quarter, followed by the MD only group and cancer & MD group. The cancer only group had the slowest increases in quarterly EOL costs, where the final month median cost was only 1.2 times that of the 6th month before death.

## Discussion

The presence of MD had a diminishing effect on utilization. Cancer patients with MD had lower costs than those without concomitant MD. This was different from expectations and contrary to results of past research looking at dollar amounts in measuring patient utilization. However, the results of this study are supported by two papers, both reporting inadequate treatment in cancer patients with MD. Davis et al. (2022), in a study examines pancreatic cancer treatment compliance in patients with prodromal depression or anxiety found that patients with prodromal depression or anxiety were significantly less likely to receive chemotherapy [30]. A second paper focused on psychiatric illness patients on colorectal cancer treatments found that individuals with a severe psychiatric illness (SPI) history had significantly less likely to receive guideline recommended treatment than colorectal cancer (CRC) patients with no history of MD. Stage II and III CRC patients with SPI history were 2.15 times less likely (95% CI 1.07–4.33) to receive potentially curative surgical resection and 2.07 times less likely (95% CI 1.72–2.50) to receive adjuvant radiation or chemotherapy [31]. Unfortunately, the reasons behind the inadequacies of treatment of cancer patients with MD cannot be interpreted directly from claims data [32]. Nonetheless, these results could indicate the adverse situation of MD patients in Taiwan in experiencing potential barriers to healthcare (non-adherence, unwillingness to seek and comply with treatment or delays in care), or a dilution effect in the treatment of MD instead of patients’ major chronic disease. In addition, authors speculate the lowered utilization of cancer patients with psychiatric morbidity could also stem from the adverse behavioral effects caused by mental disorder. To have a deeper understanding of how mental disorder negatively impacts adherence, we turn to studies directly assessing correlates of mental disease with patient attitudes toward treatment. Past research revealed 3 major views held by patients associated with MD, which acted as barriers to healthcare; first inadequate information about treatment or misinterpretation of the treatment [33]; secondly, fears about treatment and its side-effects [34]; third, forgetting appointments [35, 36].

Cancer patients with a pre-existing MD had lower expenditures than those without any concomitant MD, while patients with MD onset post-cancer had higher expenditures. This difference in sequence order effect could be caused by the pre-existing mental distress affecting access to treatment in patients during the period immediately after cancer diagnosis, or simply a longer time period with MD in pre-cancer MD patients. Another recent paper on seniors with prostate cancer found that those with prior severe mental illness had lower likelihood of receiving treatment in the first year after diagnosis [37]. For example, the adjusted odds ratio of undergoing surgery was 0.66 (95% CI: 0.49–0.89) and receiving radiation concurrent with hormone therapy was 0.81 (95% CI: 0.67–0.98). Pre-existing mental distress has been found to profoundly affect both delay in treatment and access to treatment, thereby potentially caused the reduced expenditures in this type of patients of this study. Another research disclosed that despite the increased mortality from cancer in people with MD patients, this population receives less cancer screening compared with that of the general population [38]. On the other hand, the set of patients that developed mental disorders post-cancer were found to have higher average annual expenditures per capita than those without MD at any time. [39]The possible reason is that MD will reduce treatment compliance, this result is similar to the previous research, and it is believed that patients with SMI are prone to palliative care; it revealed that SMI patients are prone to palliative care [32]. This also explains why we found that patients in the Post-Cancer MD group were the highest risk to become HUs.

In addition, a research pointed that HU is the group that accounts for the majority of healthcare spending, even disclosed that < 1% of HU in the US account for 22% of US healthcare spending [40]. We found that the costs in "Free of Both" group began to increase after Q3, therefore, we further analyze the time cost trend of EOL. An earlier analysis of Taiwan NHI expenditures comparing EOL costs of various chronic illnesses ranked cancer second in terms of highest final year EOL costs for decedents, but it did not look at disease specific cost trends approaching the final quarters or months [20]. Instead, it analyzed changes in type of medical expense and found that final quarter costs of decedents were dominated by acute inpatient expenses for most chronic diseases. However, other studies focused on cancer patients showed that cancer end-of-life care usually involved palliative or hospice care (ORs ranging 1.79 to 6.88); lower likelihood of intensive care at EOL (ORs ranging 0.26–0.68); lower odds of chemotherapy near death (ORs 0.41, 0.57); lower odds of emergency department use and shorter length of hospital stay [41]. Another research also indicated that individuals with bipolar disorder were more likely to receive palliative care and less likely to receive high-intensity EOL care [42]. Conversely, "Free of both" group had more EOL costs because of progressively complex medical needs [43, 44].

## Conclusion

Using a nationally representative database of the population of Taiwan, the study evaluated the expenditures of cancer patients with concomitant mental disorders, with focus on comparing the effects of differences in the sequence of disease onset. Contrary to expectations, the presence of mental disorder could have a diminishing effect on utilization, specifically if the onset of mental disorder was prior to cancer. Patients with post-cancer mental disorders and cancer patients without MD had the highest and second highest costs among groups, respectively. The additional expenditures of cancer patients later diagnosed with concomitant mental disorder may come from more office-visits for mental health care, and excess medication. [45, 46] Patients diagnosed with cancer and pre-existing mental illness had lower expenditures compared to those with only cancer, indicating possible barriers to care. A pre-existing mental disorder reduced expenditures in cancer patients, potentially due to barriers to uptake of treatment caused by psychiatric distress in the period immediately after cancer diagnosis. Those with pre-cancer MD had lower average expenditures, lowered risk of high utilization than patients with post-cancer MD. To the author’s best knowledge, this is the first study to analyze the effects of cancer and mental disorder diagnosis sequence differences on medical expenditure in Taiwan, and the first to distinguish pre & post-cancer, pre-cancer and post-cancer patient group differences in the same study population.

### Limitations

Research limitations of this study include; the main source of data is from the NHIRD Database and does not include certain patient variables such as lifestyle factors that potentially influences medical utilization; although lymph node and tumor size, and individually prescribed treatments are available in the database, these variables would not be applicable to all cancers included in the sample; cost data only includes NHIRD reimbursement claims and does not include patient self-pay amounts for additional treatment not covered under the NHIRD; premium-based salary as categorized by the NHIRD database only includes income as reported to the NHIRD Administration for premium calculation and may not reflect true household income; the region reported in the NHIRD database may not be the actual region of utilization as those covered under Taiwan’s national insurance are free to seek treatment nationwide.

## Supplementary Information

**Additional file 1: Figure S1.** Study populations flow chart. **Table S1.** Number of Patients by Cohort & Cancer Type. **Table S2.** HU status by cancer cohort.

## Data Availability

The data that support the findings of this study are available from the Health and Welfare Data Science Center, but restrictions apply to the availability of these data, which were used under license for the current study, and so are not publicly available. Data are however available from the authors upon reasonable request and with permission of Health and Welfare Data Science Center.

## Abbreviations

**ACPP**: Annual cost per patient
**CCI**: Charlson comorbidity index
**CRC**: Colorectal cancer
**EOL**: End-of-life
**GLM**: Generalized linear model
HWDC: Welfare data science center
**HU**: High-utilizers
**MD**: Mental disorder
**MOHW**: Ministry of health and welfare
**NHIRD**: National health insurance research database
PSM: Propensity score matching
SPI: Severe psychiatric illness
VIF: Variance inflation factor
WHO: World health organization
