# Supplementary material for: Disparities in the medical expenditures of patients with cancer and concomitant mental disorder: analyzing the effects of diagnosis sequence order
Source: BMC Health Serv Res. 2023 Jan 27;23:92. doi: 10.1186/s12913-023-09056-9 (PMC9881331; doi:10.1186/s12913-023-09056-9)
Supplement: Supplementary file 1 — Additional file 1: Figure S1. Study populations flow chart. Table S1. Number of Patients by Cohort & Cancer Type. Table S2. HU status by cancer cohort. [file 12913_2023_9056_MOESM1_ESM.docx]

**Supplementary Information**

**2005-2015 Data Science Center Database**

[N=2,000,000]

**Exclude: minors (age < 20years)**, **year 2005-2006, unconfirmed diagnosis (only 1 instance) and patients with missing data**

[N=1,286,421]

**PSM matched**

[321,133]

**Cancer and Mental Disorder**

**Exclude duration of disease < 1 year**

[321,045]

**Figure S1.** Study populations flow chart

| **Table S1.** Number of Patients by Cohort &Cancer Type | | | | |  |  |  |  |
| --- | --- | --- | --- | --- | --- | --- | --- | --- |
| **Cohort** | **N** | **%** |  | **Cancer** | **N** | **%** | **MD** | **Prevalence** |
| Pre & Post-Cancer MD | 2,498 | 0.39% |  | Lung | 2,471 | 12.70% | 1,036 | 42% |
| Pre-Cancer MD | 2,847 | 0.44% |  | Liver | 2,747 | 14.10% | 1,105 | 40% |
| Post-Cancer MD | 2,182 | 0.34% |  | Colorectal | 3,435 | 17.60% | 1,261 | 37% |
| Cancer Only | 11,963 | 1.86% |  | Breast | 3,577 | 18.40% | 1,441 | 40% |
| MD Only | 301,555 | 46.96% |  | Oral | 3,048 | 15.60% | 1,072 | 35% |
| Free of Both | 321,133 | 50.01% |  | Other | 4,212 | 21.60% | 1,612 | 38% |
|  |  |  |  | **Total** | **19,490** | **100%** | **4,846** | **39%** |

| **Table S2.** HU status by cancer cohort | | | | |
| --- | --- | --- | --- | --- |
| **Cohort** | | **N** | **HU** | **HU %** |
|  | Pre & Post-Cancer MD | 2,498 | 100 | ***4%*** |
|  | Pre-Cancer MD | 2,847 | 142 | ***5%*** |
|  | Post-Cancer MD | 2,182 | 284 | ***13%*** |
|  | Cancer Only | 11,963 | 1,107 | ***9%*** |
